# Aberrant c-AMP signalling in richter syndrome revealed by single-cell transcriptome and 3D chromatin analysis

**DOI:** 10.1186/s40364-024-00723-5

**Published:** 2025-01-23

**Authors:** Heng Li, Cheng Xing, Ji Li, Yihao Zhan, Ming Luo, Peilong Wang, Yue Sheng, Hongling Peng

**Affiliations:** 1 Department of Hematology, The Second Xiangya Hospital, Central South University, Changsha, 410011 Hunan China; 2 Institute of Molecular Hematology, Central South University, Changsha, 410011 Hunan China; 3 Hunan Engineering Research Center of Cell Immunotherapy for Hematopoietic Malignancies, Changsha, 410011 Hunan China; 4 Department of General Surgery, The Second Xiangya Hospital, Central South University, Changsha, 410011 Hunan China

**Keywords:** Richter syndrome, Chronic lymphocytic leukaemia, scRNA-seq, Chromosome conformation capture sequencing, Hi-C, cAMP-mediated signalling

## Abstract

Richter syndrome (RS), characterized by aggressive lymphoma arising from chronic lymphocytic leukaemia (CLL), presents a poor response to treatment and grim prognosis. To elucidate RS mechanisms, paired samples from a patient with DLBCL-RS were subjected to single-cell RNA sequencing (scRNA-seq) and high-throughput chromosome conformation capture (Hi-C) sequencing. Over 10,000 cells were profiled via scRNA-seq, revealing the comprehensive B cell transformation in RS. Hi-C sequencing exposed a unique chromatin architecture in RS, with increased proximal and decreased distal interactions. At the compartment scale, the interaction between B compartments was strengthened in DLBCL cells, while topologically associating domains (TADs) in DLBCL had elevated intra-TAD and reduced inter-TAD contacts. Differentially expressed genes at TAD borders between CLL and DLBCL cells highlighted an enrichment of cAMP-mediated signalling. To substantiate the functional relevance of ATF1 and CAP1, the genes involve in cAMP-mediated signalling, in the context of cell proliferation, we have performed gain- and loss-of-function experiments in relevant cell lines. Collectively, integrated scRNA-seq and Hi-C data suggest that chromatin reorganization and altered cAMP signalling drive RS transformation.

**Supplementary Information:**

The online version contains supplementary material available at 10.1186/s40364-024-00723-5.

**To the editor**

Richter syndrome (RS) is a severe progression of chronic lymphocytic leukaemia (CLL) into aggressive lymphoma, with a poorly understood molecular basis and an urgent need for new treatments [1]. The application of single-cell RNA sequencing (scRNA-seq) has yielded numerous insights into the genetic events of lymphoma. Within the genome, three-dimensional chromatin structures are dynamic and could play a crucial role in transcriptional regulation.

We integrated scRNA-seq and high-throughput chromosome conformation capture (Hi-C) sequencing to analyze a patient with CLL that transformed into diffuse large B-cell lymphoma (DLBCL). After RS diagnosis, paired samples of peripheral blood and lymph node tissue were collected (Fig. 1A; Supplemental Methods).

**Fig. 1:**
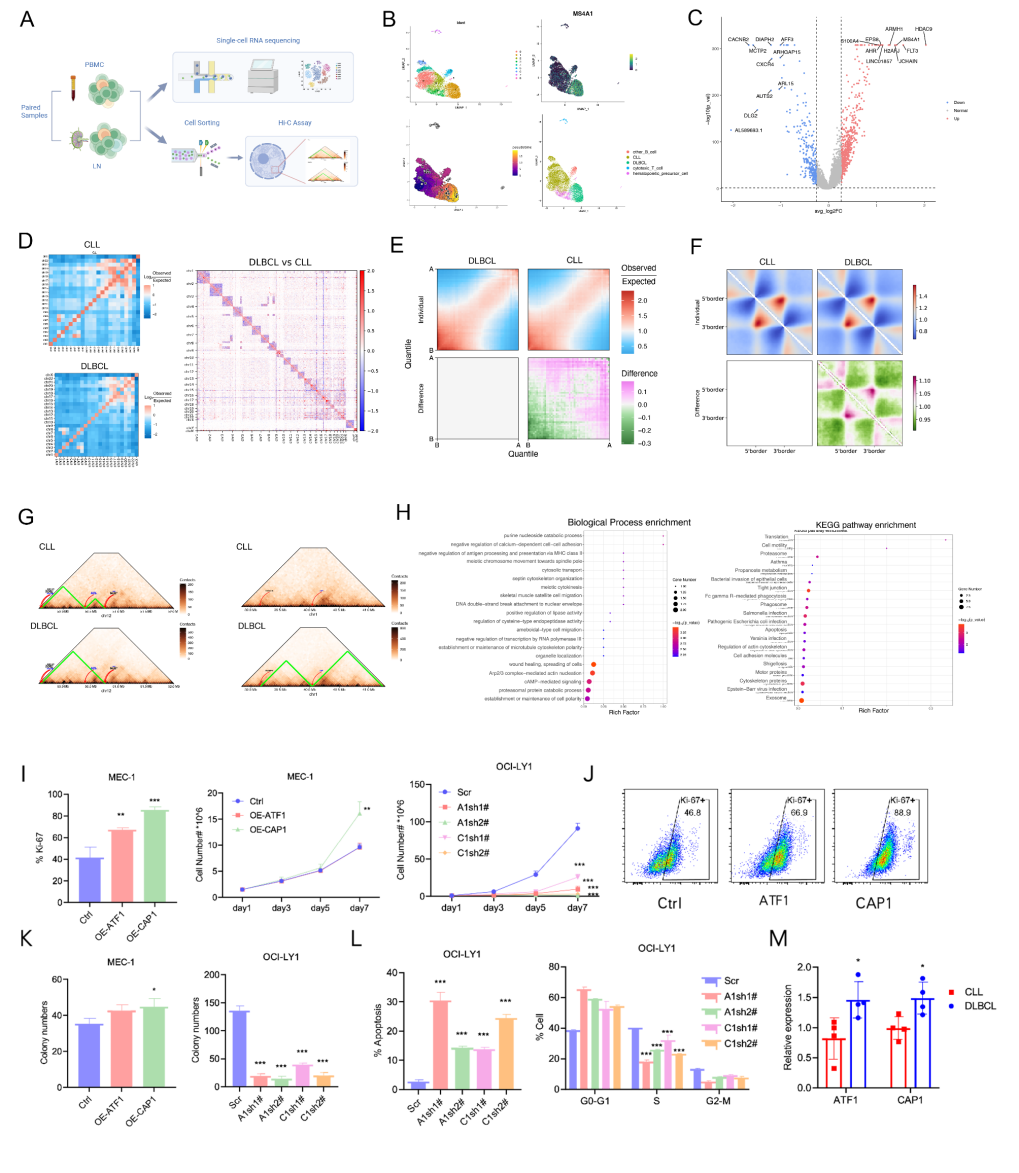
Richter’s syndrome (RS) transform undergoes global chromatin reorganization. **A** Overview of the study design. CLL cells of peripheral blood mononuclear cells and the ‘Richter transformed’ lymphonodus tissue of same patient were analyzed for cell type-specific transcriptional changes by single-cell RNA-seq (scRNA-seq), and for chromatin structure analysis by Hi-C sequencing. Figure created with BioRender.com. **B** Cell expression profiles were computationally clustered by nearest-neighbor relationships. Left upper: UMAP plot of cells with color-coded by different clusters. Left bottom: Pseudotime analysis defined by Monocle3. Right upper: UMAP showing the expression of MS4A1 (CD20) in all clusters. B cell clusters 0/2/4/6 with low expression levels of CD20 were identified as CLL populations; cluster 1 with a higher expression level of CD20 was identified as the DLBCL population. Right bottom: Clusters were separated into tissue compartments based on expression of cell-specific markers. **C** Volcano plot showing differentially expressed genes (DEGs) of B cells between CLL and RS samples. Top 10 up-regulated genes are marked with gene names. Each dot represents a DEG. Red color indicates up-regulated and blue indicates down-regulated. **D** Heatmaps showing the normalized Hi-C interaction frequencies of the whole genome (1 Mb bin) of CLL and DLBCL cells (left) and differential interactions between them (right). **E** Saddle plots of Hi-C data binned at 100 kb resolution showing the compartmental interactions for DLBCL cells (top left) and CLL cells (top right), and the differential interactions between them (bottom right). **F** Aggregation analysis for TADs showing the interactions for CLL (top left) and DLBCL (top right) and differential interactions between them (bottom right), wherein green denotes loss of interactions and red denotes gain of interactions in DLBCL cells. **G** TAD fusion for chromosome 12 (49.5–52.0 Mb) and chromosome 1 (39.0-41.5 Mb) in CLL and DLBCL cells. **H** Top 20 enriched GO biological process terms (left) and top 20 enriched KEGG pathway terms (right) for differentially expressed genes in B cells between RS and CLL samples which were located in merged TADs. **I** The CLL cell line MEC1, with overexpression of ATF1 or CAP1, displays variations in Ki-67 labeling index and cell number growth compared to the control group (Ctrl). In the DLBCL cell line OCI-LY1, the growth of cells with knockdown of ATF1 or CAP1 compared to the scramble control. **: *P* < 0.01; ***: *P* < 0.001. **J** Flow cytometry analysis of Ki-67 expression in MEC1 CLL cell line with overexpression of ATF1 or CAP1 compared to the Ctrl. **K** Comparative analysis of colony numbers in MEC1 cells with Ctrl and overexpressing ATF1 or CAP1; and in OCI-LY1 cells with scramble and knockdown of ATF1 or CAP1. *: *P* < 0.05; ***: *P* < 0.001. **L** Apoptosis and cell cycle status in OCI-LY1 cells with scramble control and knockdown of ATF1 or CAP1. ***: *P* < 0.001. **M** Real-time PCR detection of ATF1 and CAP1 mRNA expression levels in PBMCs from four CLL patients and lymph node tissues from four patients transformed into DLBCL

Overall, 10,434 single-cell transcriptomes passed quality control (Table S1) and three major cell types were identified: B cells, cytotoxic T cells and haematopoietic precursor cells (Fig. S1). Six clusters of B cells were identified (Fig. 1B, left upper panel). Fig. S2 shows the top 4 differentially expressed genes in each cell cluster. According to the pseudotime trajectories (Fig. 1B, left lower panel) and the expression level of CD20 in B cells (Fig. 1B, right upper panel), CLL cells were mostly mapped to Clusters 0, 2, 4 and 6; RS B cells were mostly mapped to Cluster 1 (Fig. 1B, right lower panel). The differentially expressed genes (DEGs) between CLL and DLBCL cells are shown in Fig. 1C. GO analysis revealed that the DEGs were enriched in immune system processes (Fig. S3). PySCENIC analysis identified top 5 master regulators of DLBCL cell clusters (Fig. S4).

B cells undergo dynamic genome architecture reorganization during differentiation and neoplastic transformation. Hi-C sequencing revealed that the interaction matrices of DLBCL cells exhibited enhanced proximal interactions and weakened distal interactions compared with those of CLL cells (Fig. 1D). DLBCL cells had increased intrachromosomal contact probabilities at < 50 Mb and decreased at > 50 Mb, with proximal contacts significantly exceeding distal ones (Figure S5).

Genomic regions can be assigned into the active compartment (compartment A) and the inactive compartment (compartment B) [2]. At the compartment level, DLBCL cells showed strengthened interaction between B compartments (StableBB) and compromised StableAA and SwitchAB (Fig. 1E). GO enrichment analysis of genes in StableAA, StableBB, SwitchAB and SwitchBA is shown in Figure S6.

At the topologically associating domain (TAD) scale, the insulation score increased in the DLBCL cells (Figure S7), with increased intra-TAD and decreased inter-TAD contacts (Figure S8), indicating that the TADs were compacted and that the interactions between adjacent TADs were inhibited. Aggregate peak analysis also revealed differential interactions between DLBCL cells and CLL cells (Fig. 1F).

We observed that 6.4% of the DEGs, including 7.0% of the upregulated genes (Table S3) and 5.4% of the downregulated genes (Table S4), were located at the TAD boundaries. The upregulated genes inside merged TADs (UPGs^TAD^) were involved in wound healing, cAMP-mediated signalling and proteasomal processes (Fig. 1H). KEGG analysis revealed that the UPGs^TAD^ were enriched in exosome and apoptosis signalling (Fig. 1H), whereas the DWGs^TAD^ were enriched in ribosome signalling (Figure S9-10). ATF1 and CAP1 are representative UPGs^TAD^ involved in cAMP-mediated signalling, the locations of which are shown in Fig. 1G.

Continuing our investigation, we quantified the mRNA levels of ATF1 and CAP1 in a cohort comprising four individuals with RS and those with CLL. The real-time PCR findings revealed a pronounced upregulation of both ATF1 and CAP1 in RS patients relative to CLL patients (Fig. 1M). This prompted us to further elucidate the functional roles of these genes. We proceeded to overexpress ATF1 and CAP1 in the CLL cell line MEC1. Flow cytometry analysis indicated that the Ki-67 proliferation marker was significantly elevated in cells with overexpression of ATF1 (ATF1-OE) and CAP1 (CAP1-OE) compared to the control (Fig. 1I-J). Subsequent proliferation and colony formation assays confirmed that the forced expression of CAP1-OE notably accelerated the proliferation rate and enhanced the colony-forming capacity of MEC1 cells (Fig. 1K-L). Encouraged by these results, we employed two unique shRNA constructs to knock down ATF1 and CAP1 in the DLBCL cell line OCI-LY1. Our results demonstrated that, compared to scramble control, the downregulation of ATF1 and CAP1 substantially hindered the proliferative capabilities (Fig. 1I-J) and colony-forming potential of OCI-LY1 cells (Fig. 1K). Moreover, this intervention promoted apoptosis and led to cell cycle arrest (Fig. 1L).

Cancer genomics and gene regulation have an emerging role in shaping our current understanding and future directions in cancer research [3, 4]. In the field of cancer research, bioinformatic analysis is essential for identifying key genetic alterations, predicting treatment responses, and uncovering potential therapeutic targets [5, 6]. Using longitudinal samples, we conducted a combined scRNA-seq and Hi-C analysis, demonstrating that RS lymphoma cells exhibited enhanced proximal interactions between chromosomes and reorganized chromatin, thus affecting cAMP-mediated signalling, which has been described to be relevant for a wide array of intracellular processes [7]. Specifically, one of the UPGs^TAD^ involved in cAMP-mediated signalling was ATF1, whose upregulation is associated with cancer proliferation and the tumour microenvironment [8]. Another UPG^TAD^ involved in cAMP-mediated signalling was CAP1, which was reported to be associated with poor prognosis in cancer [9].

The potential mechanisms by which chromatin reorganization influences gene expression and cell function in RS are complex. Firstly, increased TAD compaction could potentially lead to altered gene expression by bringing enhancers and promoters into closer spatial proximity, thereby affecting the transcriptional output [10]. Meanwhile, histone modifications and DNA methylation patterns may accompany changes in chromatin-remodeling [11].

Our study is the first to explore the chromatin-reorganization-gene regulation link in RS. We propose that it is worthwhile to identify how the fusion of TADs in transformed DLBCL cells results in transcriptome alterations. Our findings highlight the aberrant cAMP signalling in RS, which suggests that pharmacological modulation of cAMP could be a potential therapeutic avenue. Specifically, inhibitors of phosphodiesterases, which are enzymes that degrade cAMP, could be explored as a means to potentially counteract the aggressive phenotype of RS [12]. Furthermore, ATF1 and CAP1 could serve as prognostic biomarkers for RS. We propose that the expression levels of these genes could be used to stratify RS patients into different risk groups.

Despite the valuable insights gained from the integrated analysis of scRNA-seq and Hi-C data, our study is not without limitations. First, the full spectrum of mechanisms underlying chromatin reorganization and gene expression changes in RS is likely to be multifaceted and complex. The potential functional roles and mechanisms of DEGs that are not related to TAD structural changes should not be overlooked. Second, the depth of the Hi-C sequencing data presented here did not reach the ideal coverage of 300X, which is considered necessary for a comprehensive exploration of chromatin structures, including the detailed analysis of loop formations. We highlight the need for future research to investigate these genes and their implications in RS pathogenesis to elucidate the complete picture.

## Electronic supplementary material

Below is the link to the electronic supplementary material.

Supplementary Material 1

Supplementary Material 2

Supplementary Material 3

Supplementary Material 4

Supplementary Material 5

Supplementary Material 6

## Data Availability

The clinical information, sc-RNA sequencing data and Hi-C sequencing data in this article are available upon request.

## Abbreviations

RS: Richter syndrome
CLL: Chronic lymphocytic leukaemia
scRNA-seq: Single-cell RNA sequencing
Hi-C: High-throughput chromosome conformation capture
DLBCL: Diffuse large B-cell lymphoma
UMAP: Uniform manifold approximation and projection
DEGs: Differentially expressed genes
TAD: Topologically associating domain
UPGs^TAD^: Upregulated genes inside merged TADs
DWGs^TAD^: Downregulated genes inside merged TADs
