# Supplementary material for: Aberrant c-AMP signalling in richter syndrome revealed by single-cell transcriptome and 3D chromatin analysis
Source: Biomark Res. 2025 Jan 23;13:15. doi: 10.1186/s40364-024-00723-5 (PMC11756191; doi:10.1186/s40364-024-00723-5)
Supplement: Supplementary file 2 — Supplementary Material 2 [file 40364_2024_723_MOESM2_ESM.pdf]

Table S2
Quality control of Hi-C sequencing

Sample Name	Raw Reads	Clean Reads	Raw Bases(G)	Clean Bases(G)	Q20(%)	Q30(%)	GC Content(%)
CLL	2174649502	2137105510	326.2	312.13	97.85	93.2	42.45
DLBCL	1756787436	1727731470	263.52	251.14	97.76	93.03	43.04
