# Supplementary material for: Aberrant c-AMP signalling in richter syndrome revealed by single-cell transcriptome and 3D chromatin analysis
Source: Biomark Res. 2025 Jan 23;13:15. doi: 10.1186/s40364-024-00723-5 (PMC11756191; doi:10.1186/s40364-024-00723-5)
Supplement: Supplementary file 3 — Supplementary Material 3 [file 40364_2024_723_MOESM3_ESM.pdf]

Table S3

List of upregulated genes inside merged TADs between CLL cells and DLBCL cells

Gene_name	Chr	Start	End	Strand	distance_To_TADBoundary	TAD_boundary	boundary_Site	p_val	pct.1	pct2.
ACTR2	chr2	65225753	65229753	+	-10247	chr2_65240000	65240000	7.67805712384644e-85	0.872	0.958
ANP32E	chr1	150234156	150238156	-	-1844	chr1_150240000	150240000	1.56573495824185e-212	0.586	0.877
ARPC5	chr1	183633757	183637757	-	-2243	chr1_183640000	183640000	3.75007268205179e-56	0.734	0.874
ATF1	chr12	50761710	50765710	+	1710	chr12_50760000	50760000	1.66488233903383e-84	0.269	0.511
CAP1	chr1	40038233	40042233	+	-1767	chr1_40040000	40040000	9.92162586510021e-50	0.777	0.909
CAPG	chr2	85416432	85420432	-	-19568	chr2_85440000	85440000	4.12810755760345e-80	0.65	0.855
CD151	chr11	830843	834843	+	-5157	chr11_840000	840000	6.70224161718704e-103	0.148	0.364
CD58	chr1	116569039	116573039	-	9039	chr1_116560000	116560000	1.00127537464109e-129	0.315	0.604
CDK2AP2	chr11	67506649	67510649	-	-9351	chr11_67520000	67520000	2.15989005352018e-70	0.514	0.744
CLECL1	chr12	9731299	9735299	-	11299	chr12_9720000	9720000	2.91984484717531e-243	0.446	0.804
DNAJC7	chr17	42019376	42023376	-	-16624	chr17_42040000	42040000	2.29859528116553e-77	0.694	0.868
EML6	chr2	54721499	54725499	+	1499	chr2_54720000	54720000	5.88537506405989e-121	0.117	0.345
FXYD5	chr19	35152730	35156730	+	-3270	chr19_35160000	35160000	1.52525878755381e-90	0.938	0.99
GADD45GIP1	chr19	12955236	12959236	-	-764	chr19_12960000	12960000	1.79519500291079e-90	0.646	0.854
HLA-DOB	chr6	32818466	32822466	-	-17534	chr6_32840000	32840000	3.57433723341249e-51	0.634	0.81
LDHB	chr12	21755857	21759857	-	-143	chr12_21760000	21760000	1.75608486183677e-45	0.786	0.913
LMNA	chr1	156080573	156084573	+	573	chr1_156080000	156080000	2.1100627773164e-45	0.336	0.519
LRCH3	chr3	197789226	197793226	+	-6774	chr3_197800000	197800000	1.45746840087873e-49	0.536	0.709
LRRFIP1	chr2	237625576	237629576	+	-10424	chr2_237640000	237640000	3.48511886118883e-46	0.668	0.825
MYL12A	chr18	3245481	3249481	+	5481	chr18_3240000	3240000	2.4311127516187e-72	0.818	0.939
NUDT1	chr7	2240222	2244222	+	222	chr7_2240000	2240000	3.15056939904674e-195	0.684	0.928
PDE8A	chr15	84978440	84982440	+	18440	chr15_84960000	84960000	4.75682934725339e-69	0.393	0.612
PLAGL1	chr6	144062599	144066599	-	-13401	chr6_144080000	144080000	3.67719905707685e-97	0.248	0.494
PRDX5	chr11	64316088	64320088	+	88	chr11_64320000	64320000	1.56849521182808e-62	0.801	0.937
PSMB8	chr6	32842703	32846703	-	2703	chr6_32840000	32840000	1.79802226185462e-73	0.744	0.903
PSMB9	chr6	32842136	32846136	+	2136	chr6_32840000	32840000	9.3239557913304e-65	0.775	0.921
RHOC	chr1	112705434	112709434	-	-10566	chr1_112720000	112720000	2.44231675647227e-59	0.624	0.829
RIPOR1	chr16	67516418	67520418	+	418	chr16_67520000	67520000	5.25519844777132e-135	0.291	0.586
RNH1	chr11	505300	509300	-	-10700	chr11_520000	520000	5.14085700901212e-78	0.804	0.956
SCAND1	chr20	35957472	35961472	-	1472	chr20_35960000	35960000	1.06024403435706e-56	0.675	0.842
SPIB	chr19	50416938	50420938	+	16938	chr19_50400000	50400000	3.93324796828151e-75	0.569	0.781
STX7	chr6	132511198	132515198	-	-4802	chr6_132520000	132520000	5.59831162834846e-60	0.708	0.865
TUBA4A	chr2	219276170	219280170	-	170	chr2_219280000	219280000	5.69364491425229e-99	0.481	0.738
UFC1	chr1	161150776	161154776	+	-5224	chr1_161160000	161160000	1.52617879048011e-49	0.829	0.938
