# Supplementary material for: Aberrant c-AMP signalling in richter syndrome revealed by single-cell transcriptome and 3D chromatin analysis
Source: Biomark Res. 2025 Jan 23;13:15. doi: 10.1186/s40364-024-00723-5 (PMC11756191; doi:10.1186/s40364-024-00723-5)
Supplement: Supplementary file 4 — Supplementary Material 4 [file 40364_2024_723_MOESM4_ESM.pdf]

Table S4
List of downregulated genes inside merged TADs between CLL cells and DLBCL cells

Gene_name	Chr	Start	End	Strand	distance_To_TADBoundary	TAD_boundary	boundary_Site	p_val	pct.1	pct2.
EIF5B	chr2	99335353	99339353	+	15353	chr2_99320000	99320000	2.73E-48	0.638	0.545
GNPTAB	chr12	101828938	101832938	-	-7062	chr12_10184000C	101840000	5.79E-20	0.773	0.772
IL2RA	chr10	6060370	6064370	-	-15630	chr10_6080000	6080000	3.57E-174	0.401	0.082
LHPP	chr10	124459834	124463834	+	19834	chr10_12444000C	124440000	2.78E-42	0.44	0.309
PMAIP1	chr18	59897948	59901948	+	17948	chr18_59880000	59880000	1.27E-113	0.808	0.698
PRRC2C	chr1	171483551	171487551	+	3551	chr1_171480000	171480000	1.79E-30	0.868	0.878
RPL10A	chr6	35466408	35470408	+	-9592	chr6_35480000	35480000	1.09E-106	0.999	1
RPL31	chr2	100999715	101003715	+	-285	chr2_101000000	101000000	5.06E-308	0.925	0.839
RPL9	chr4	39456949	39460949	-	16949	chr4_39440000	39440000	3.75E-90	0.996	0.997
SCMH1	chr1	41240154	41244154	-	154	chr1_41240000	41240000	1.66E-09	0.579	0.57
SF1	chr11	64776786	64780786	-	16786	chr11_64760000	64760000	1.26E-82	0.932	0.927
TESC	chr12	117097479	117101479	-	-18521	chr12_11712000C	117120000	2.92E-44	0.596	0.486
TMEM71	chr8	132758712	132762712	-	-1288	chr8_132760000	132760000	4.65E-91	0.253	0.054
UBB	chr17	16378798	16382798	+	18798	chr17_16360000	16360000	1.17E-32	0.917	0.915
UBXN4	chr2	135739619	135743619	+	-16381	chr2_135760000	135760000	2.10E-86	0.741	0.636
