# Supplementary material for: Aberrant c-AMP signalling in richter syndrome revealed by single-cell transcriptome and 3D chromatin analysis
Source: Biomark Res. 2025 Jan 23;13:15. doi: 10.1186/s40364-024-00723-5 (PMC11756191; doi:10.1186/s40364-024-00723-5)
Supplement: Supplementary file 5 — Supplementary Material 5 [file 40364_2024_723_MOESM5_ESM.docx]

**Supplemental Methods**

**Tissue preparation and flow cytometric cell sorting**

The fresh tissues were stored in phosphate buffered saline (PBS) on ice after the surgery within 30 mins. The specimens were minced into small pieces, and then digested with dissociation solution. Afterwards, the red blood cell lysis buﬀer (RCLB) was added to remove red blood cells. The PBMCs were isolated by density gradient centrifugation using Ficoll-Paque Plus medium and washed with PBS. After removed red blood cells, the PBMCs were resuspended by PBS to obtain a single-cell suspension. The single-cell suspension from lymph nodes tissue and PBMC was sorted by flowcytometry. FSC^high^ and CD20^+^ cells from lymph nodes were sorted as Richter transformed aggressive B cells; while FSC^low^ and CD20^dim^ cells from PBMC were sorted as indolent CLL cells. Finally, the samples were stained with Trypan Blue and the cell viability was evaluated microscopically.

**Single cell RNA sequencing**

Single-cell suspensions with PBS were loaded onto microwell chip using the Single Cell Processing System. Barcoding Beads are subsequently collected from the microwell chip, followed by reverse transcription of the mRNA captured by the Barcoding Beads and to obtain cDNA, and PCR amplification. The amplified cDNA is then fragmented and ligated with sequencing adapters. The scRNA-seq libraries were constructed according to the protocol of the RNA Library Kits^1^. Individual libraries were diluted to 4 nM, pooled, and sequenced on Illumina novaseq 6000 with 150 bp paired end reads. After quality control, scRNA-seq data was analysed for dimension-reduction, clustering, differentially expressed genes (DEGs) analysis, trajectory analysis, pathway enrichment analysis and transcription factor regulatory network analysis according to the published protocol^2^.

**Hi-C assay and analysis**

## Hi-C library preparation

Hi-C libraries were constructed according to previous studies^3^. Briefly, samples were cross-linked with 1% formaldehyde for 10 min at room temperature and quenched with 0.125M final concentration glycine for 5 min. The cross-linked cells were subsequently lysed. Endogenous nuclease were inactivated with 0.3% SDS, then chromatin DNA were digested by 100U MboI (NEB), and marked with biotin-14-dCTP (Invitrogen) and then ligated by 50U T4 DNA ligase(NEB). After reversing cross-links, the ligated DNA was extracted through QIAamp DNA Mini Kit (Qiagen) according to manufacturers’ instructions. Purified DNA was sheared to 300- to 500-bp fragments and were further blunt-end repaired, A-tailed andadaptor-added, followed by purification through biotin-streptavidin–mediated pull-down and PCR amplification. Finally, the Hi-C libraries were quantified and sequenced on the Illumina Nova-seq platform (San Diego, CA, USA).

**Hi-C Matrix analysis**

The differential Hi-C matrix was build using hicCompareMatrices, a tool of HiCExplorer (v3.7.3) software suite, under 10kb resolution, ‘--clearMaskedBins’ was set to remove empty bins. Contact probability was calculated using cooltools(stable version)’s subcommand expected_cis. When calcultating PvD, following the definition of short-range/long-range of HiC-Pro, contacts < 20kb was defined as ‘proximal’，contacts > 20kb was defined as ‘distal’, then count the number of proximal and distal contacts on each chromosome.

**TAD calling**

We defined TAD boundaries by insulation score analysis^4^. We calculated insulation score using 40 kb bin size and 800 kb sliding window size (average size of TAD). The insulation score was normalized by the mean of all bins per chromosome. Valleys along the normalized insulation score indicate the reduced Hi-C contact. The valleys were detected by the finding horizontal zero point of delta vector. We filtered the horizontal zero points at peaks. Boundaries with distance less than 40 kb were merged into one. plot the TAD calling results using R script and GENOVA. APA plot around TAD domains was generated by coolpuppy. First using submodule ‘coolpup.py’ with parameter ‘--local --rescale’ to produce pileup data in ‘clpy’ format, then using it as input, conducting submodule ‘plotpup.py’ with ‘--scale log’ to generate APA plot.

**GO and KEGG enrichment analysis**

GO Analysis: Gene ontology (GO) analysis was performed to facilitate elucidating the biological implications of unique genes in the significant or representative profiles of the gene in the experiment^5^. We downloaded the GO annotations from NCBI (http://www.ncbi.nlm.nih.gov/), UniProt (http://www.uniprot.org/) and the Gene Ontology (http://www.geneontology.org/). Fisher’s exact test was applied to identify the significant GO categories and FDR was used to correct the p-values.

Pathway analysis was used to find out the significant pathway of the genes according to KEGG database. We turn to the Fisher’s exact test to select the significant pathway, and the threshold of significance was defined by P-value and FDR^6^.

**Cell culture**

MEC1 and OCI-LY1 were cultured in IMDM with 10% FBS. 293T was cultured in DMEM with 10% FBS. All cells were cultured at 37 °C with 5% CO2.

**Plasmid construction and cell infection**

The human ATF1 and CAP1 genes were efficiently cloned into the PCDH vector. Primers for four distinct human shRNA constructs were annealed and then subcloned into the pLKO.1 vector. For lentiviral production, the target plasmid was co-transfected with packaging plasmids psPAX2 and pMDG.2 into 293T cells utilizing polyethylenimine (PEI). Viral supernatants were collected at 24, 48, and 72 hours following transfection. The cells were incubated with the viral medium, supplemented with 5 μg/ml polybrene, and underwent spinfection for 3 hours at 32 °C.

**Colony-forming assay**

1000 Cells were mixed with Human Methylcellulose Base Media and after 10 days, colony number was counted.

**Flow cytometric analysis**

For apoptosis detection, cells were subjected to Annexin V and DAPI staining. Regarding BrdU cell cycle analysis, cells were treated with 30 μg/ml BrdU. Following a 3-hour incubation, the cells were harvested and stained with anti-BrdU antibodies along with DAPI. For Ki-67 detection of cell proliferation, cells are fixed with 70% ethanol at -20°C and stained with a Ki-67 antibody. Subsequently, all samples were analyzed using flow cytometry.

**RT-PCR**

RT-PCR were performed as described previously^7^.

**Statistical analysis**

To determine statistical significance, a one-way ANOVA test was utilized to analyze mean differences among various groups. The data presented are expressed as means ± standard deviation (SD) and are based on a minimum of three independent experimental replicates. Significance was set at the P < 0.05.

**Supplemental References:**

1 Dura, B. *et al.* scFTD-seq: freeze-thaw lysis based, portable approach toward highly distributed single-cell 3' mRNA profiling. *Nucleic Acids Res* **47**, e16, doi:10.1093/nar/gky1173 (2019).

2 Li, H. *et al.* Clonal architecture and single-cell transcriptome landscape in Richter's syndrome. *British journal of haematology* **202**, 1055-1060, doi:10.1111/bjh.18952 (2023).

3 Rao, S. S. *et al.* A 3D map of the human genome at kilobase resolution reveals principles of chromatin looping. *Cell* **159**, 1665-1680, doi:10.1016/j.cell.2014.11.021 (2014).

4 Dixon, J. R. *et al.* Topological domains in mammalian genomes identified by analysis of chromatin interactions. *Nature* **485**, 376-380, doi:10.1038/nature11082 (2012).

5 Ashburner, M. *et al.* Gene ontology: tool for the unification of biology. The Gene Ontology Consortium. *Nature genetics* **25**, 25-29, doi:10.1038/75556 (2000).

6 Draghici, S. *et al.* A systems biology approach for pathway level analysis. *Genome Res* **17**, 1537-1545, doi:10.1101/gr.6202607 (2007).

7 Sotillo, E. *et al.* Convergence of Acquired Mutations and Alternative Splicing of CD19 Enables Resistance to CART-19 Immunotherapy. *Cancer Discov* **5**, 1282-1295, doi:10.1158/2159-8290.Cd-15-1020 (2015).
