# Supplementary material for: Aberrant c-AMP signalling in richter syndrome revealed by single-cell transcriptome and 3D chromatin analysis
Source: Biomark Res. 2025 Jan 23;13:15. doi: 10.1186/s40364-024-00723-5 (PMC11756191; doi:10.1186/s40364-024-00723-5)

**Supplementary Figures**

**Fig. S1 UMAP plot of cells with color-coded by different clusters.**


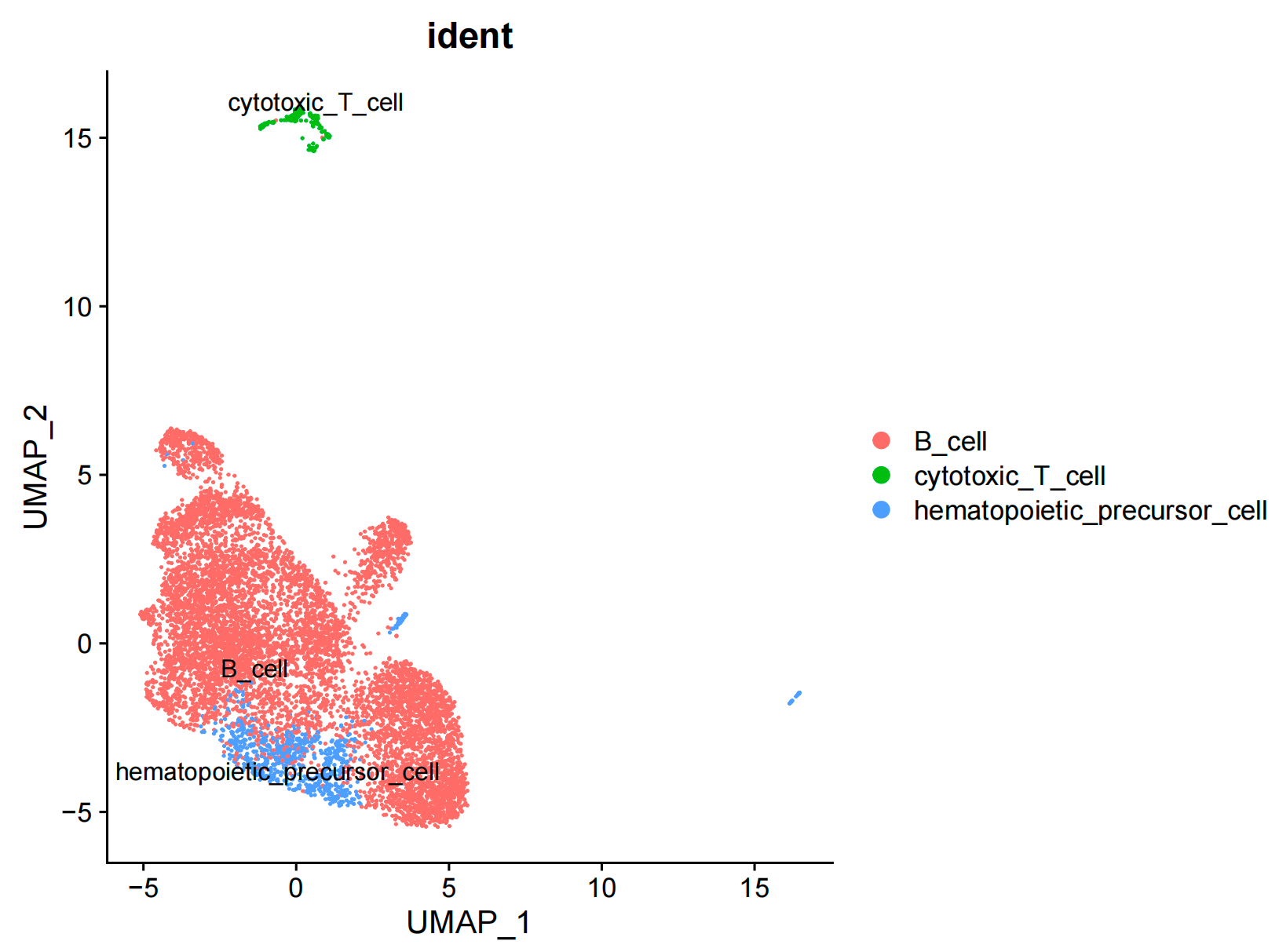


**Fig. S2 Dot plot showing top 4 differentiated genes of cell clusters. Each row represents a cluster. The colour indicates the expression levels of genes.**


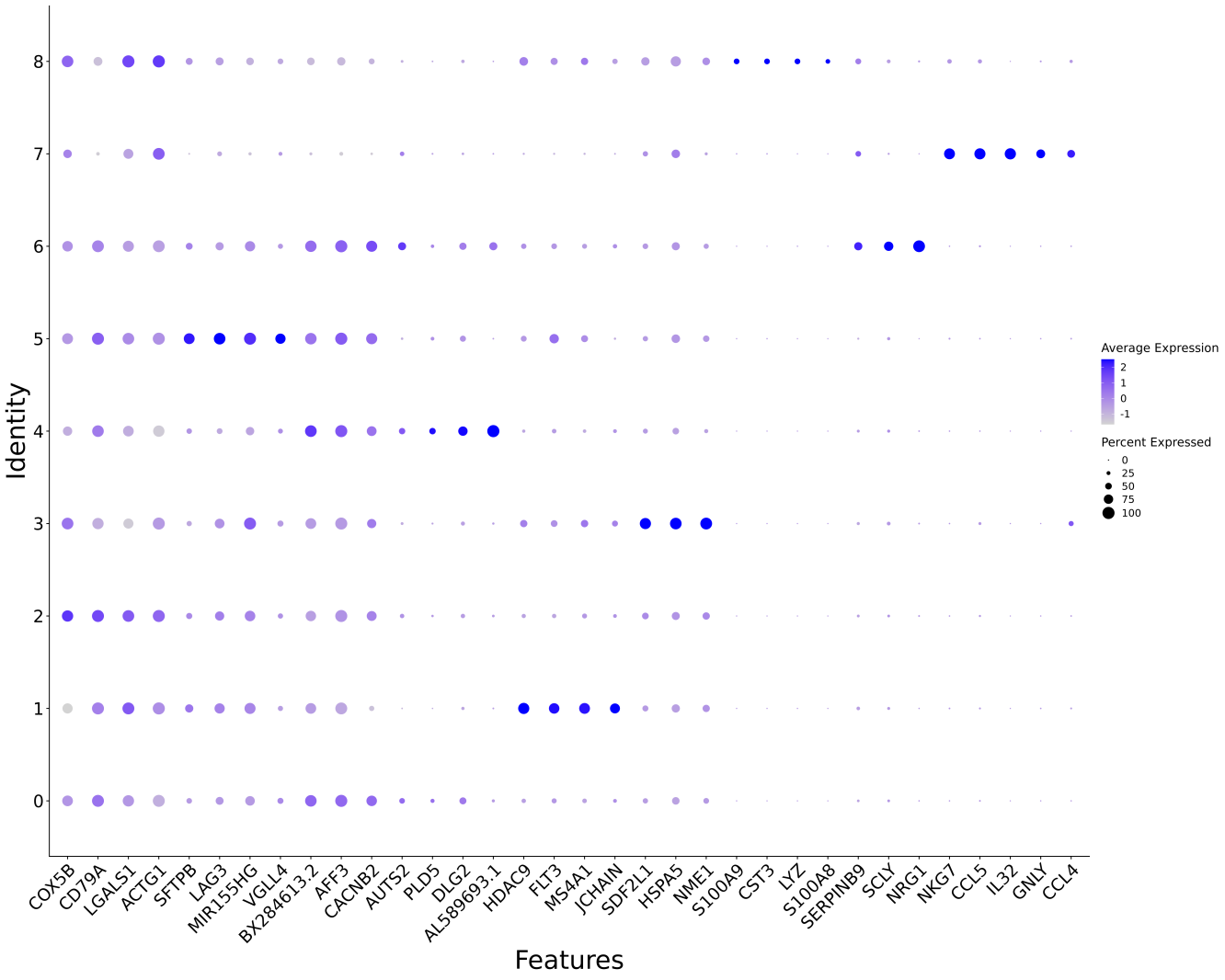


**Fig. S3 Top 20 enriched GO biological process terms for differentially over-expressed genes (A) and down regulated genes (B) in B cells between RS and CLL samples.**

(A)


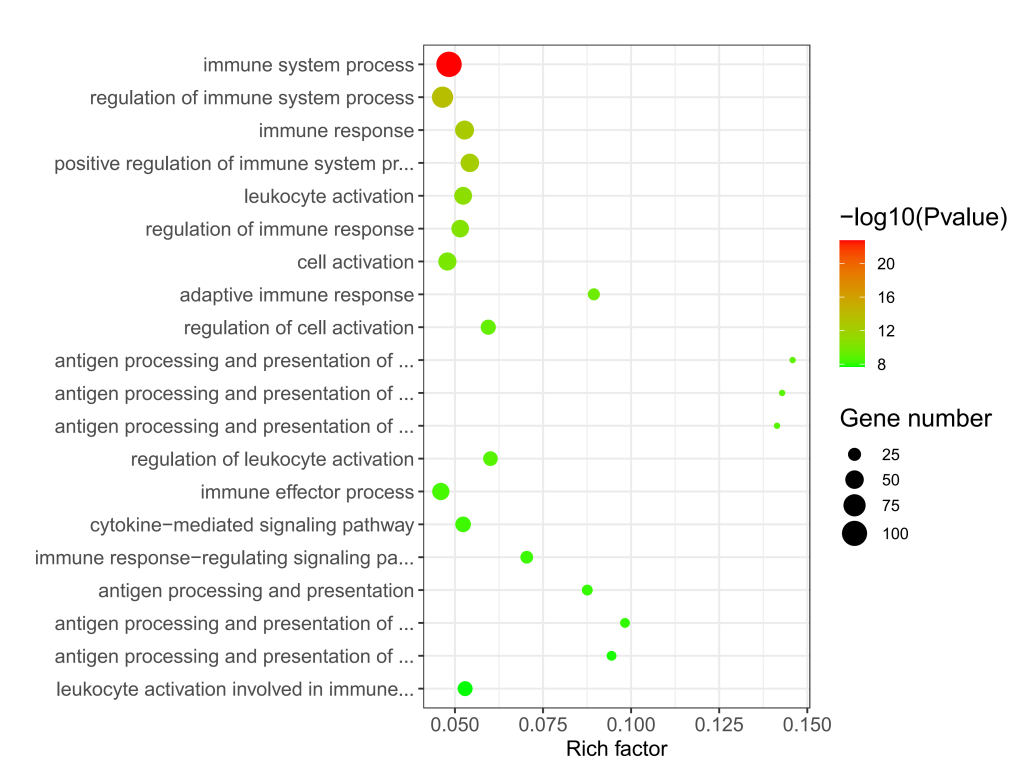


(B)


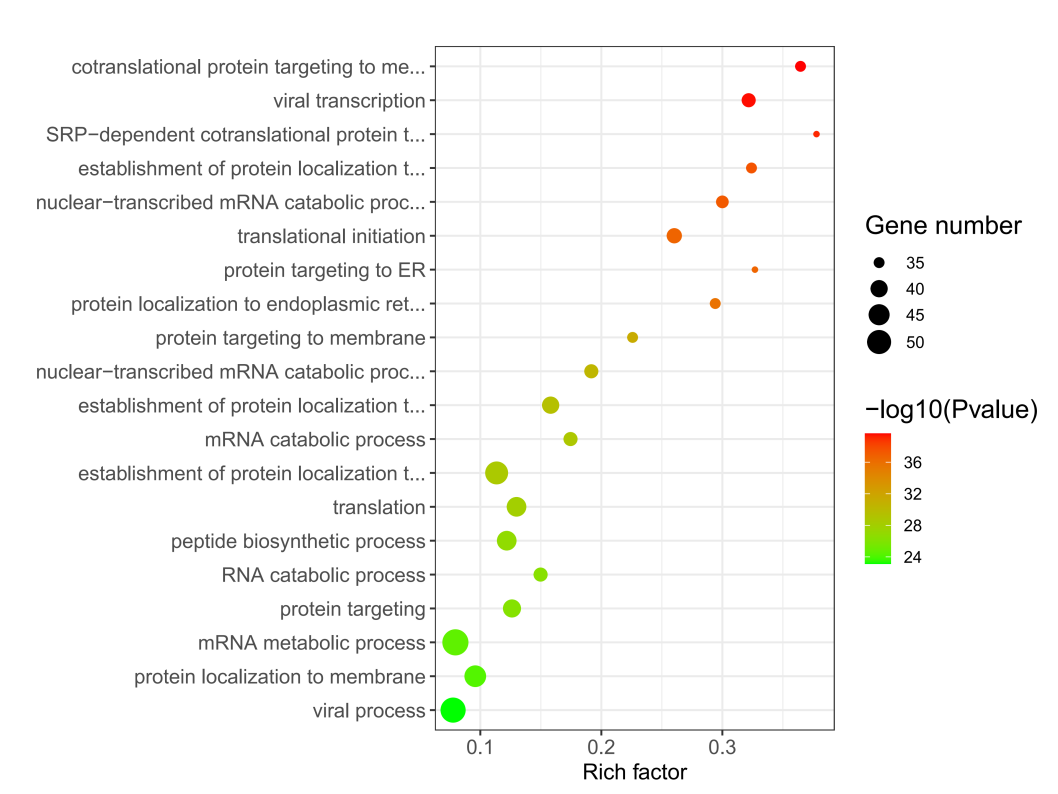


**Fig. S4 Top 5 regulons in the DLBCL cell cluster by pySCENIC analysis.**


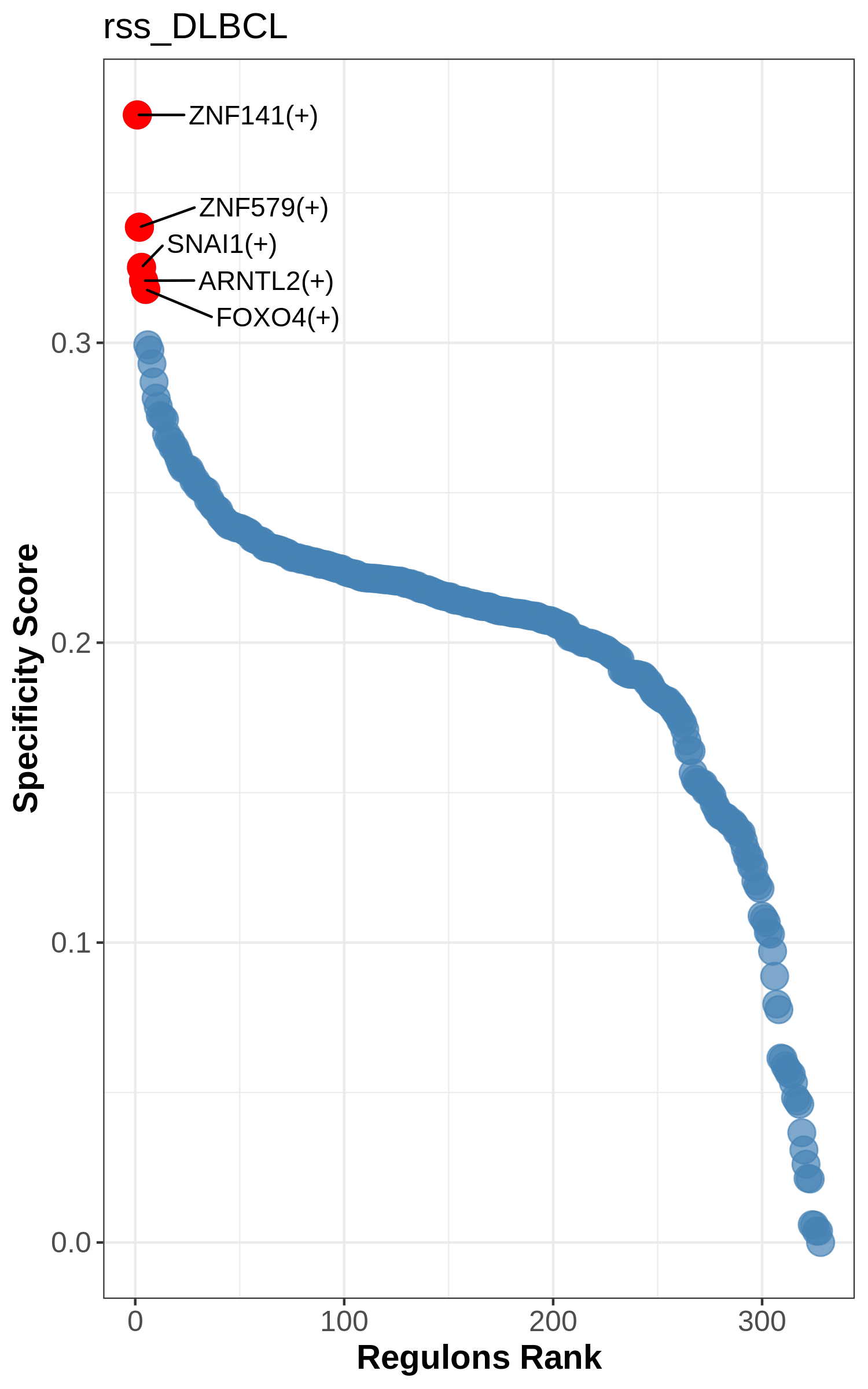


**Fig. S5 The intrachromosomal contact probability of CLL and DLBCL cells. Intrachromosomal contact probability refers to the likelihood of any two loci (regions of interest) on the same chromosome coming into physical proximity within the three-dimensional space of the nucleus.**

1. **The decaying curve shows the genome-wide contact probability for interactions within individual chromosomes by genomic distance. The x-axis stands for the genomic distance (Mb) and the y-axis stands for the contact probability. The dotted line represents 50 Mb. DLBCL cells had increased intrachromosomal contact probabilities at <50 Mb and decreased at >50 Mb.**


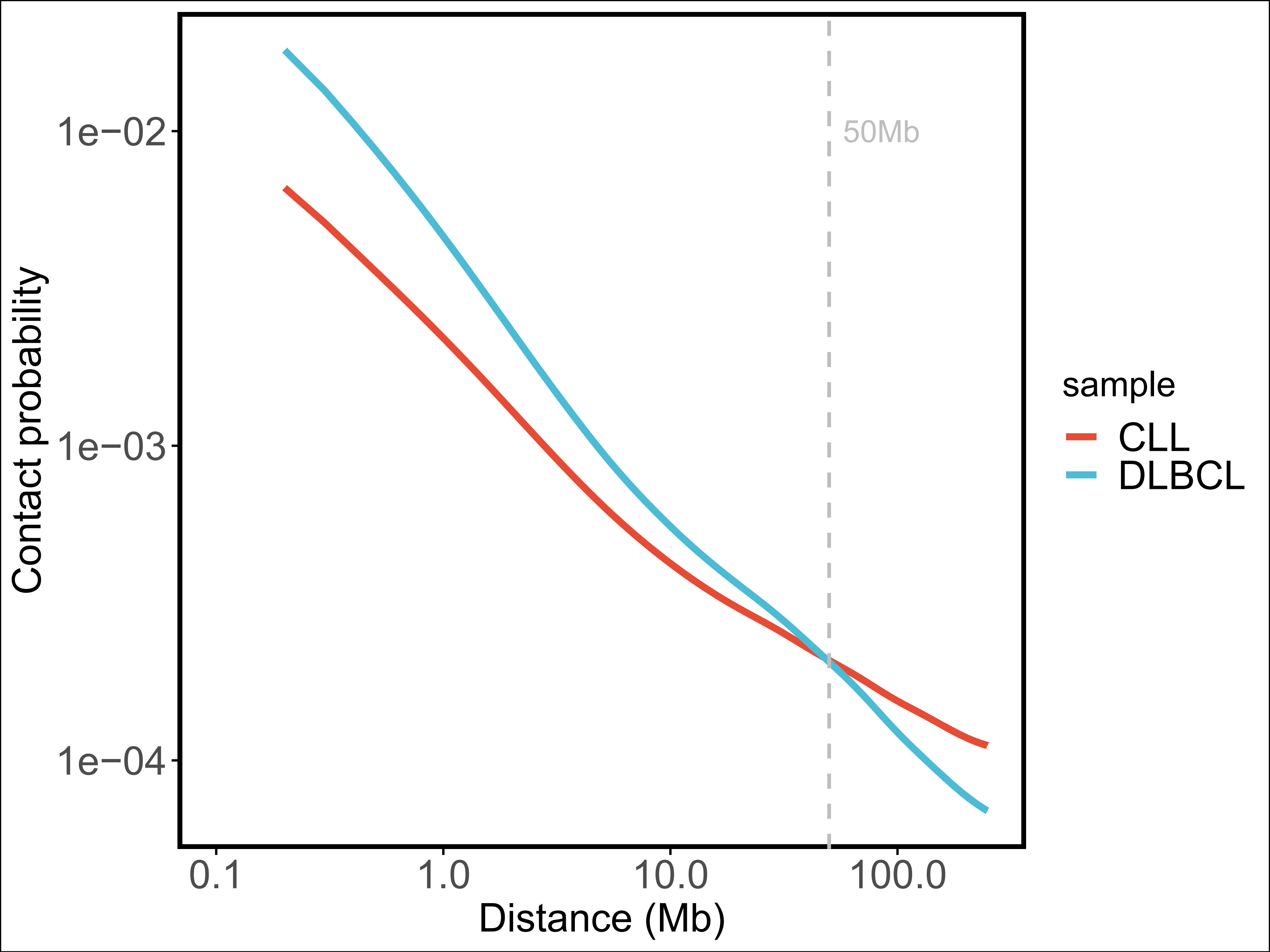


1. **The boxplot showing the ratio of proximal and distal contacts (PvD) for CLL and DLBCL cells. The DLBCL sample has a significantly increased PvD ratio than CLL. ****: P< 0.0001.**


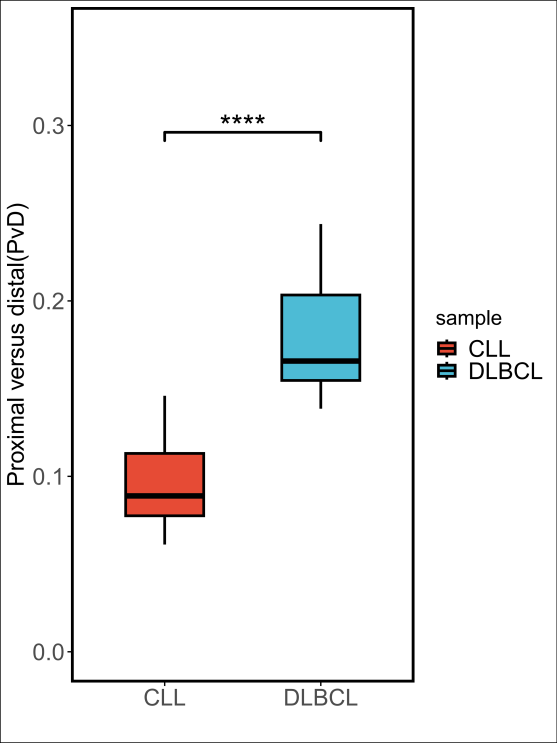


**Fig. S6 Top 20 enriched GO biological process terms for genes in StableAA (A), StableBB (B), SwitchAB (C) and SwitchBA (D).**

(A)


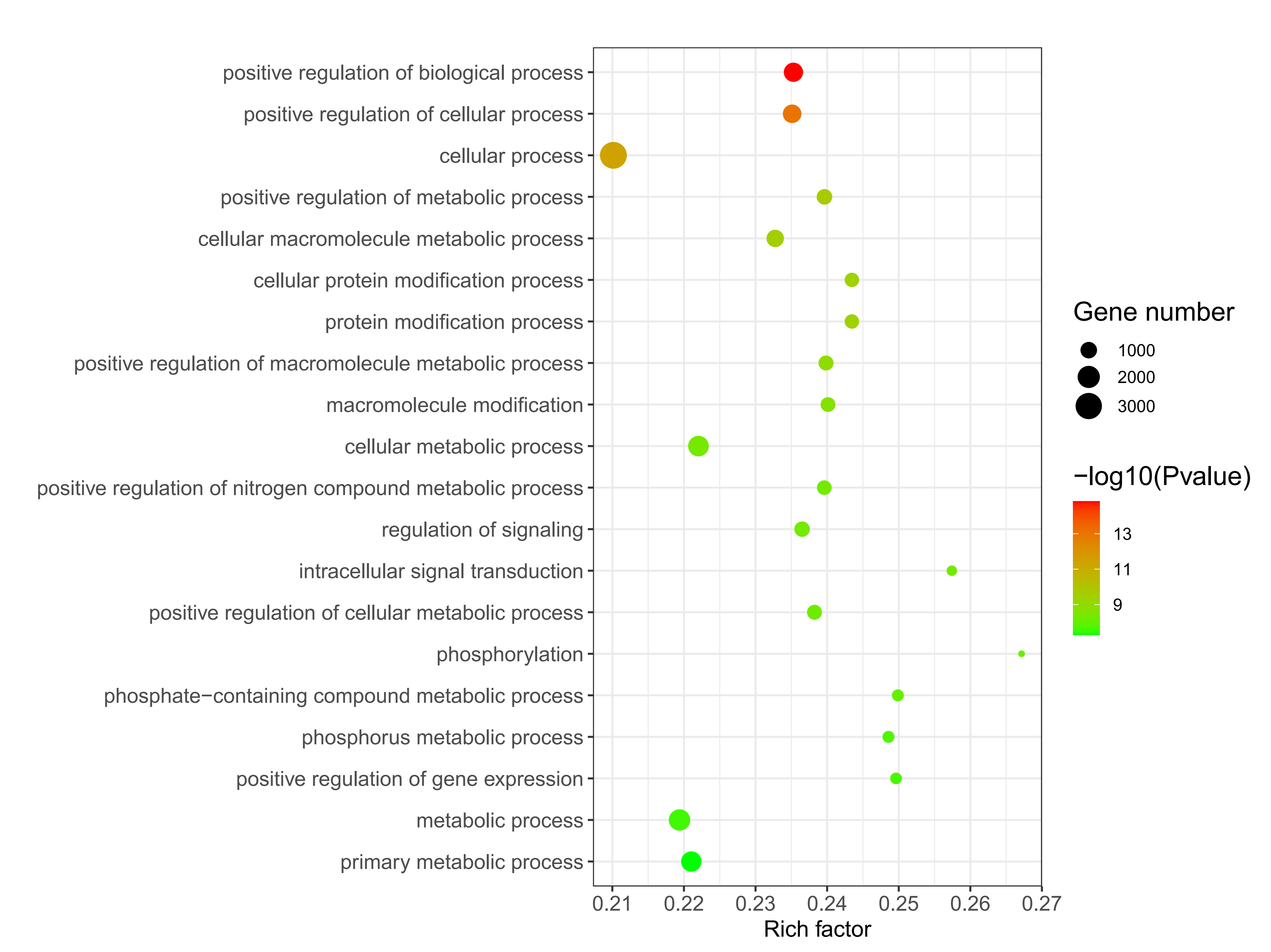


(B)


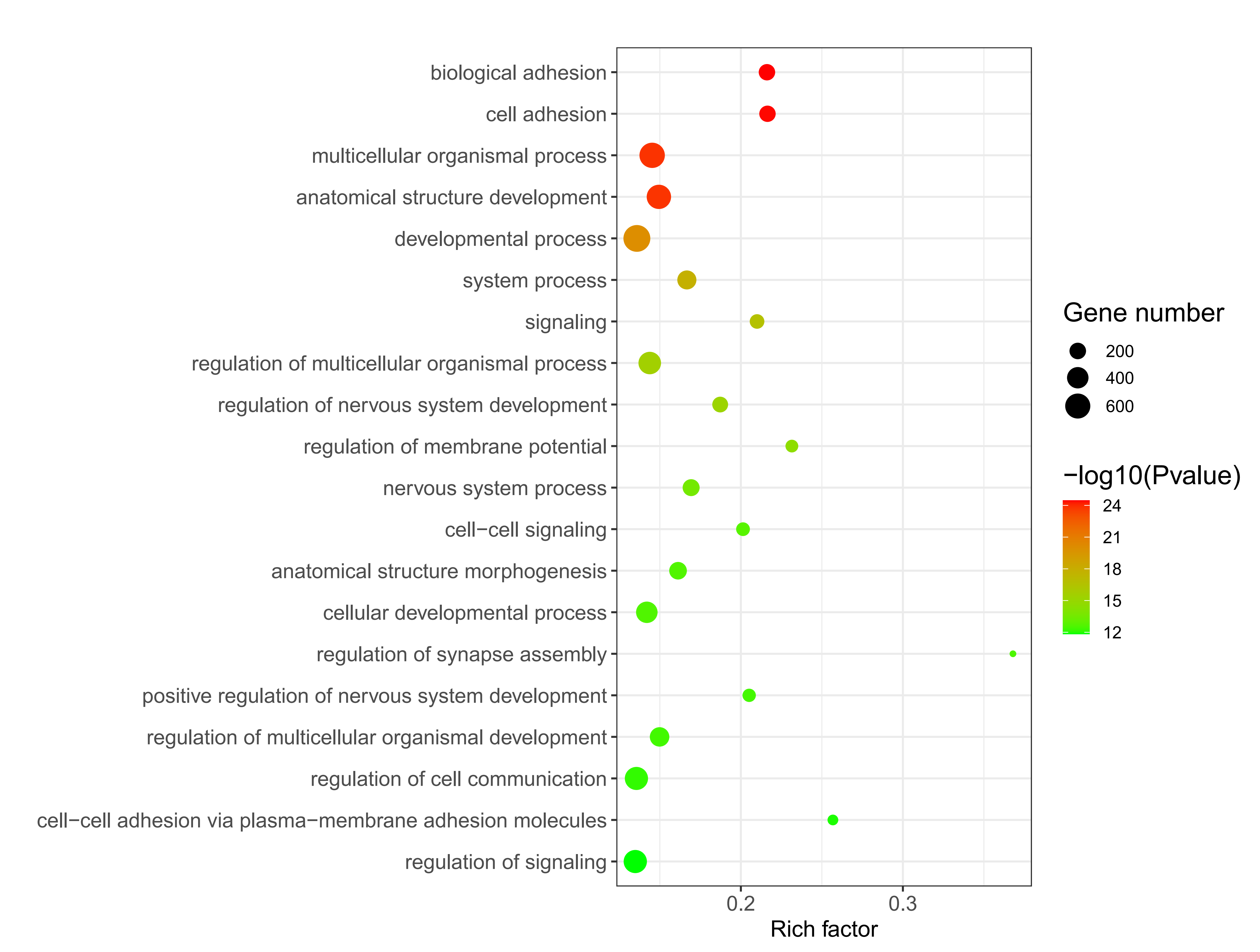


(C)
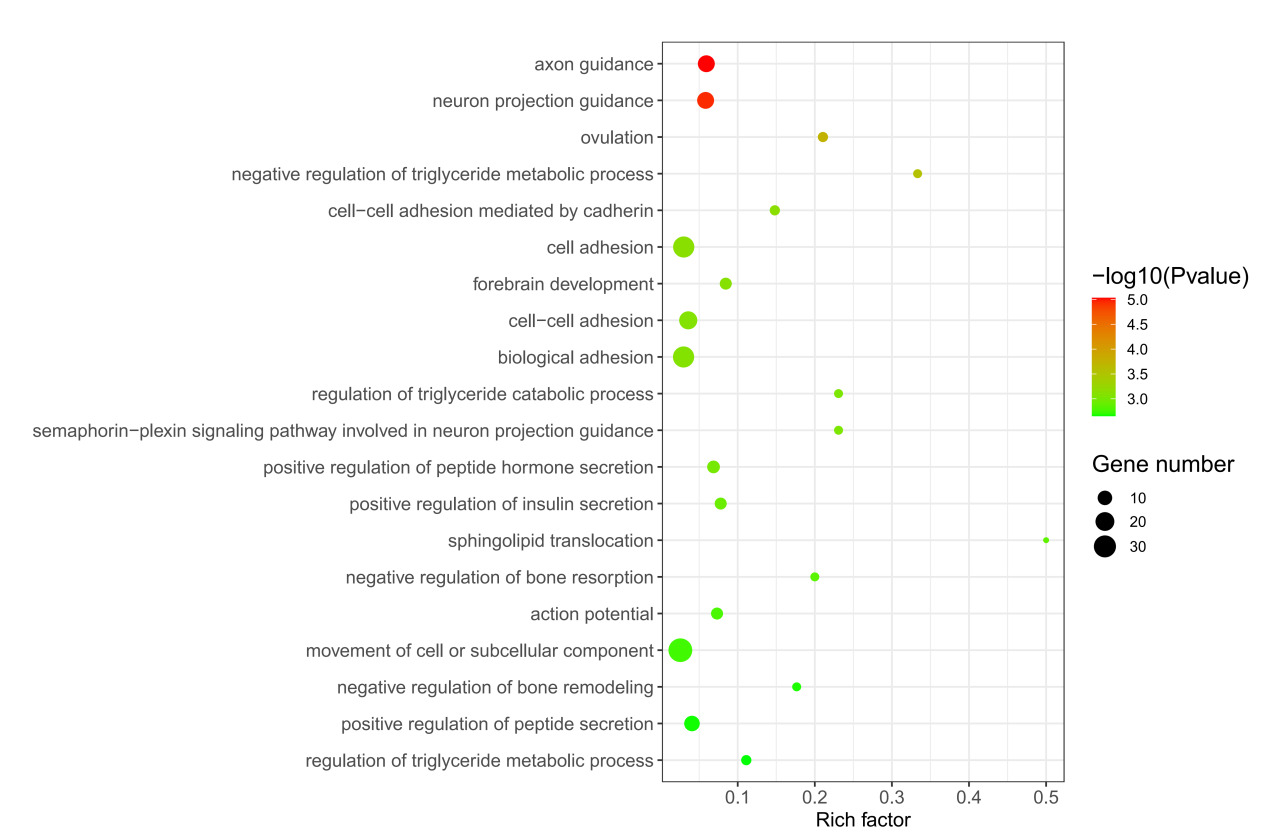


(D)


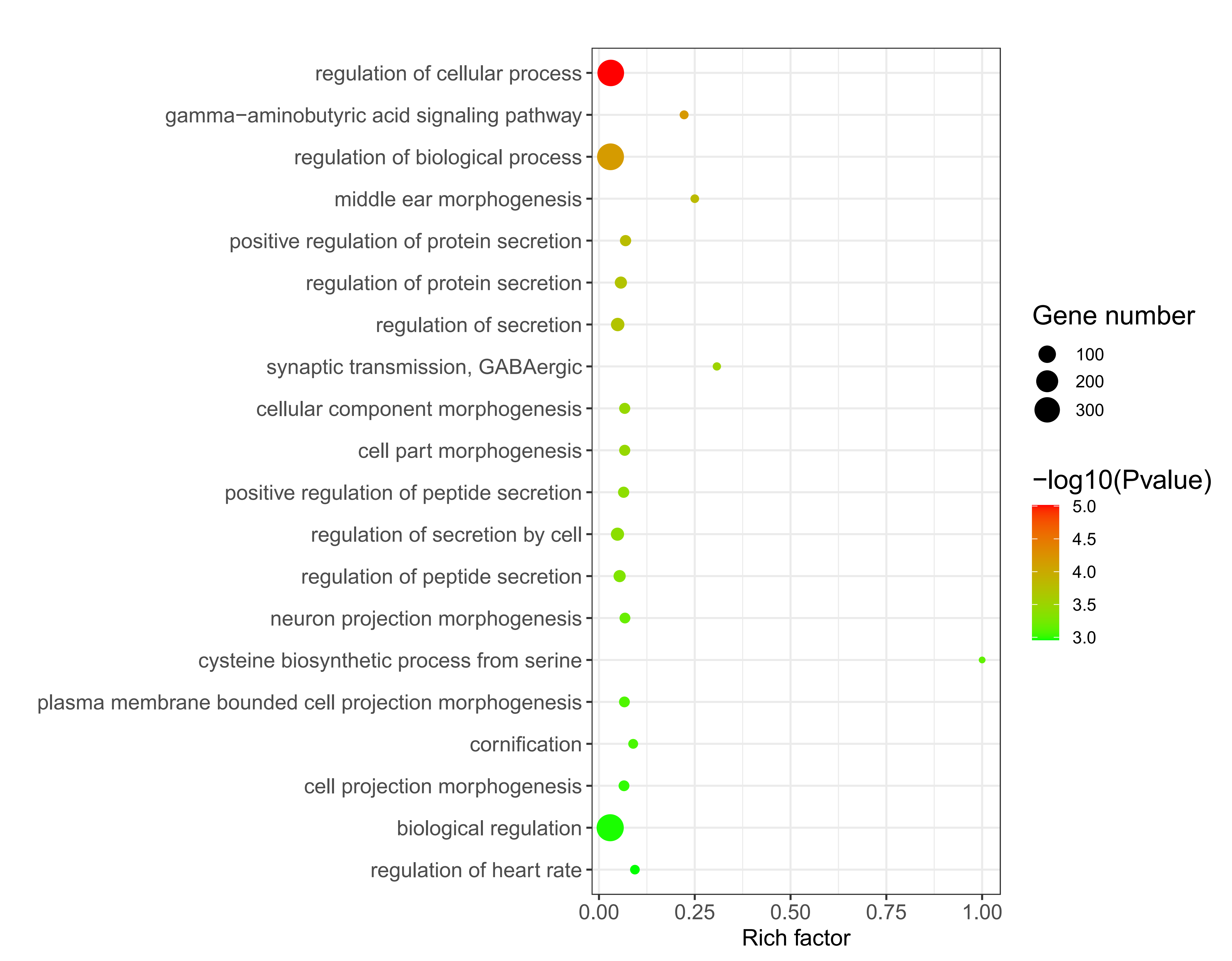


**Fig. S7 These curves show the insulation score around TAD boundaries in CLL and DLBCL cells (±1 Mb).**


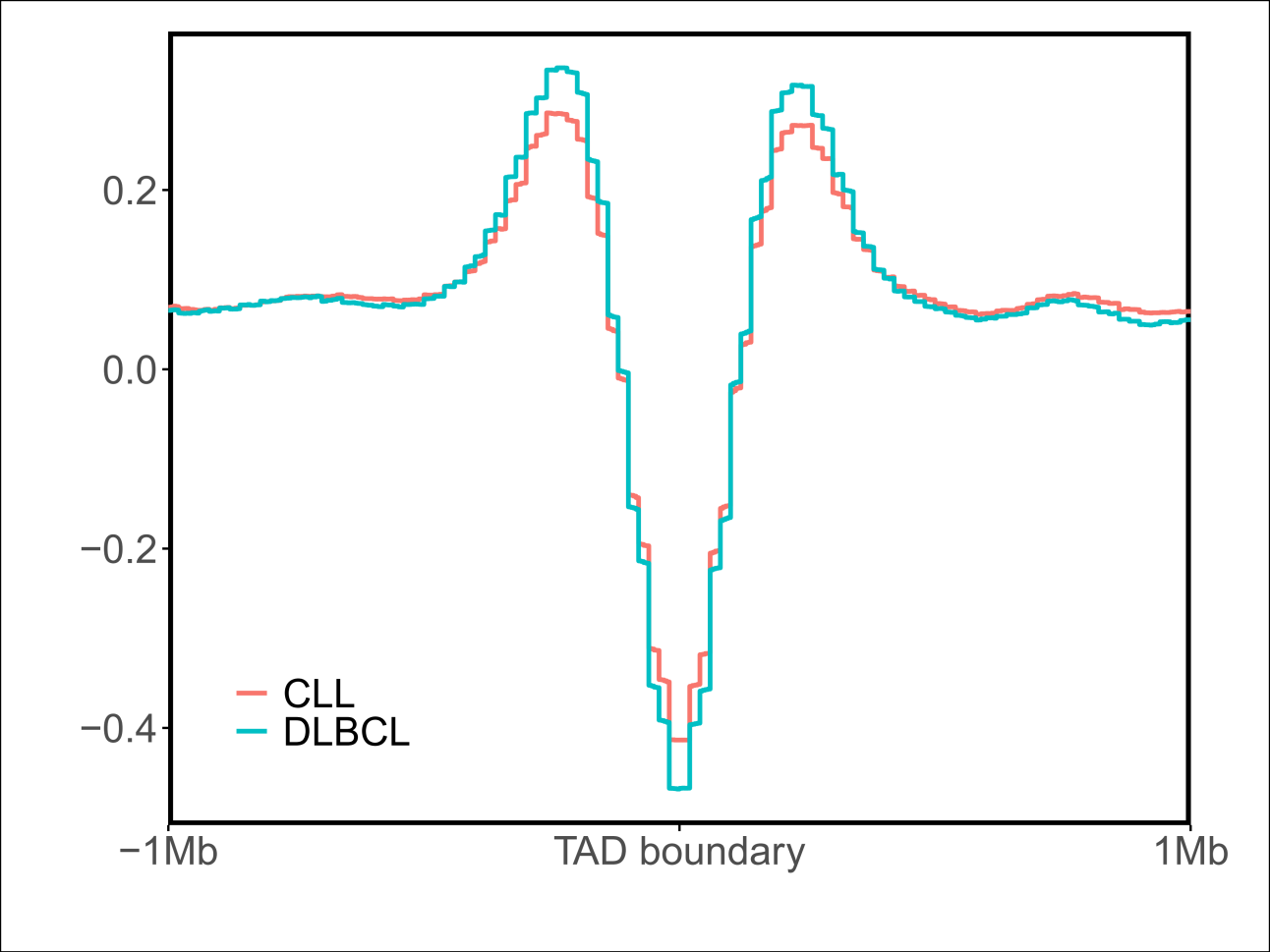


**Fig. S8 These boxplots show the contact probability of intra-TAD and inter-TAD interactions for CLL and DLBCL cells. **, P<0.01; ****, P<0.0001.**


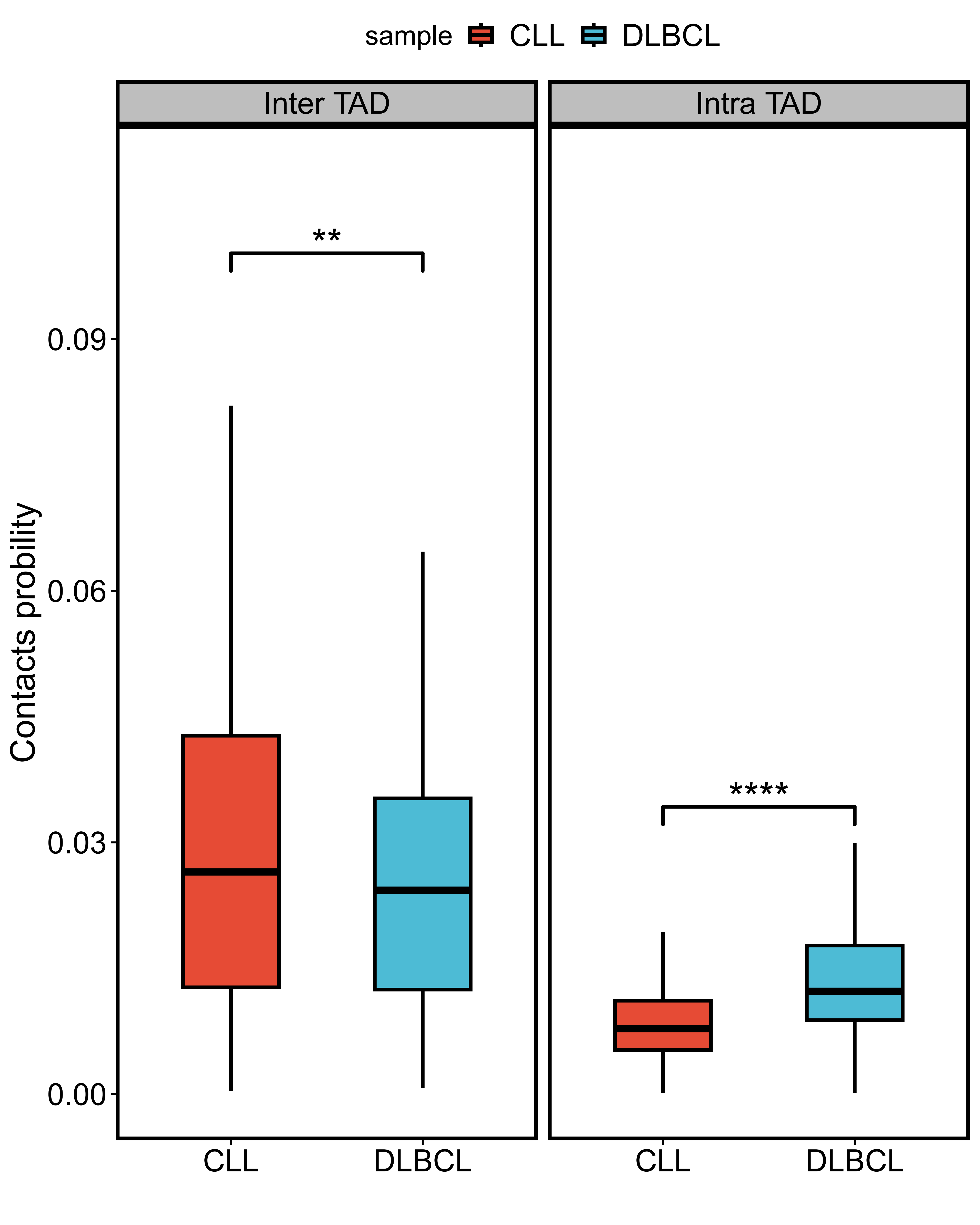


**Fig. S9 Top 20 enriched GO biological process terms for differentially down-regulated genes in B cells between RS and CLL samples which were located in merged TADs.**


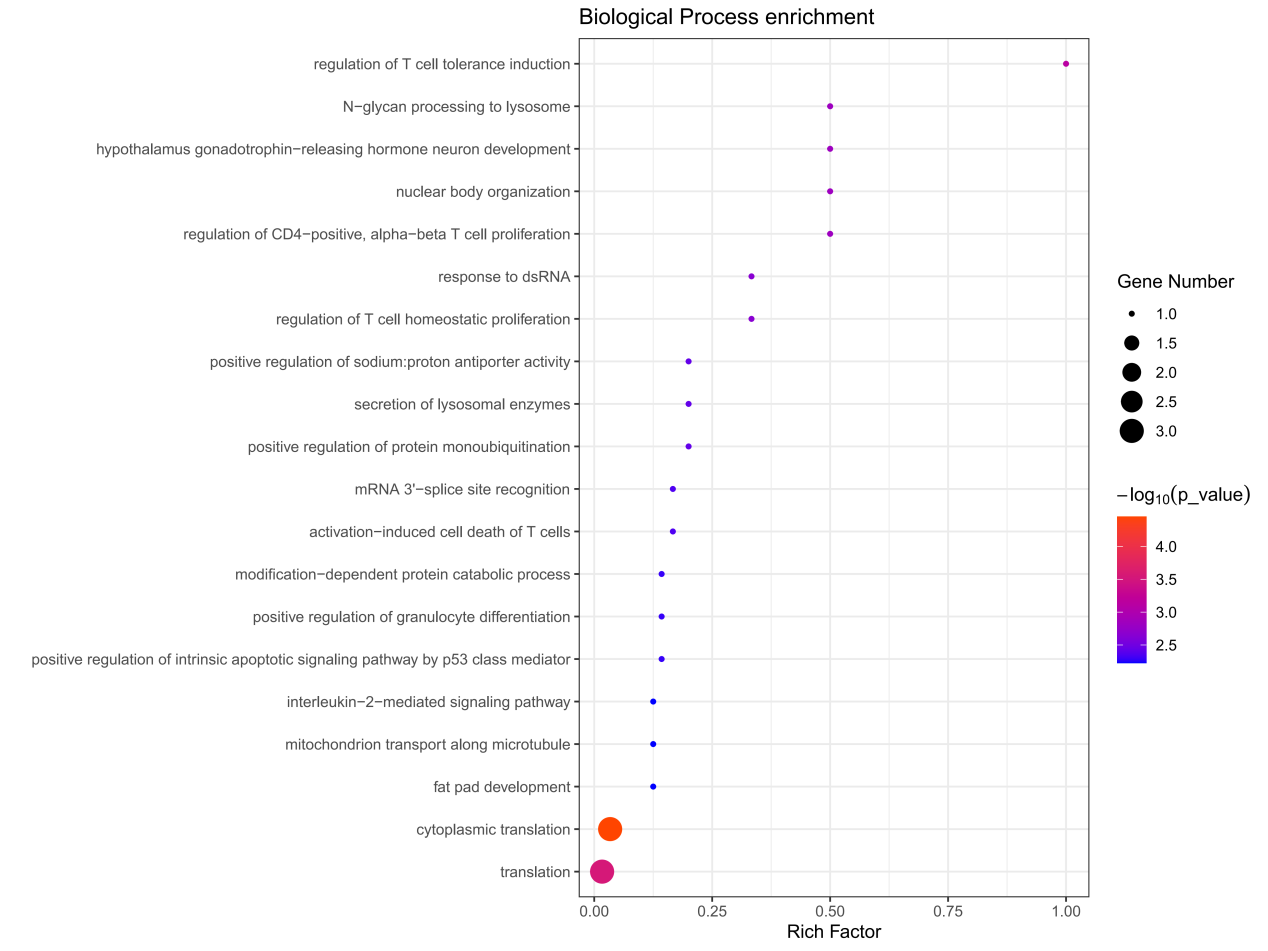


**Fig. S10 Top 20 enriched KEGG pathway terms for differentially down-regulated genes in B cells between RS and CLL samples which were located in merged TADs.**


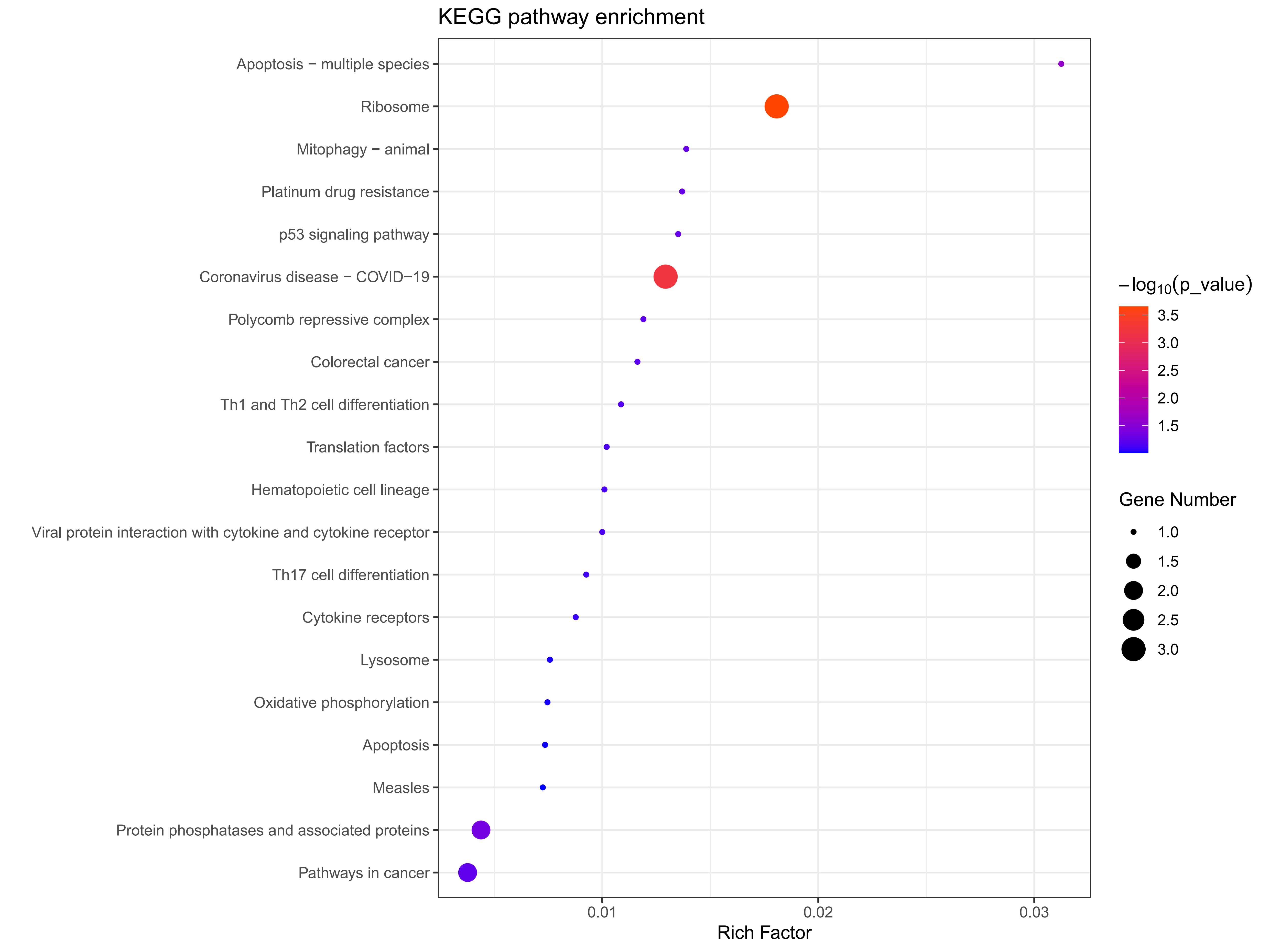

Supplement: Supplementary file 6 — Supplementary Material 6 [file 40364_2024_723_MOESM6_ESM.docx]
